# Supplementary material for: A UMLS-based spell checker for natural language processing in vaccine safety
Source: BMC Med Inform Decis Mak. 2007 Feb 12;7:3. doi: 10.1186/1472-6947-7-3 (PMC1805499; doi:10.1186/1472-6947-7-3)
Supplement: Additional file 13 — RAP application directory. Sets up directory for RAP (RDF application for PHP) source code [file 1472-6947-7-3-S13.gz › rap/doc/tests/test_infmodel.html]

### RDF API for PHP V0.9.1

# Test: Using the InfModel

  
This example is part of the RAP - Rdf API for PHP documentation.  
  
Setting up the model:

//change the RDFAPI\_INCLUDE\_DIR to your local settings  
define("RDFAPI\_INCLUDE\_DIR", "C:/!htdocs/rdfapi-php/api/");   
include(RDFAPI\_INCLUDE\_DIR . "RdfAPI.php");

//create a InfmodelF  
$infModel= ModelFactory::getInfModelF('http://InfModelF.org');

**Added the following Triples:**  
<http://example.org/baz1> <http://example.org/bat> <http://example.org/baz2> .  
<http://example.org/bat> <http://www.w3.org/2000/01/rdf-schema#subPropertyOf> <http://example.org/bas> .

//add new statements  
$infModel->add(new Statement(new Resource('http://example.org/baz1'),  
                                           new Resource('http://example.org/bat'),  
                                           new Resource('http://example.org/baz2')));  
  
$infModel->add(new Statement(new Resource('http://example.org/bat'),  
                                           new Resource('http://www.w3.org/2000/01/rdf-schema#subPropertyOf'),  
                                           new Resource('http://example.org/bas')));

//output the model as HTML table  
$infModel->writeAsHtmlTable();

  

|  |  |  |  |
| --- | --- | --- | --- |
| **Base URI:** http://InfModelF.org# | | | **Size:** 3 |
| **No.** | **Subject** | **Predicate** | **Object** |
| 1. | Resource: http://example.org/baz1 | Resource: http://example.org/bat | Resource: http://example.org/baz2 |
| 2. | Resource: http://example.org/bat | RDF Node: http://www.w3.org/2000/01/rdf-schema#subPropertyOf | Resource: http://example.org/bas |
| 3. (infered) | Resource: http://example.org/baz1 | Resource: http://example.org/bas | Resource: http://example.org/baz2 |

  

---

  
**Added the following Triples:**  
<http://example.org/bat> <http://www.w3.org/2000/01/rdf-schema#domain> <http://example.org/Domain1> .  
<http://example.org/bat> <http://www.w3.org/2000/01/rdf-schema#range> <http://example.org/Range1> .
  

|  |  |  |  |
| --- | --- | --- | --- |
| **Base URI:** http://InfModelF.org# | | | **Size:** 7 |
| **No.** | **Subject** | **Predicate** | **Object** |
| 1. | Resource: http://example.org/baz1 | Resource: http://example.org/bat | Resource: http://example.org/baz2 |
| 2. | Resource: http://example.org/bat | RDF Node: http://www.w3.org/2000/01/rdf-schema#subPropertyOf | Resource: http://example.org/bas |
| 3. (infered) | Resource: http://example.org/baz1 | Resource: http://example.org/bas | Resource: http://example.org/baz2 |
| 4. | Resource: http://example.org/bat | RDF Node: http://www.w3.org/2000/01/rdf-schema#domain | Resource: http://example.org/Domain1 |
| 5. (infered) | Resource: http://example.org/baz1 | RDF Node: http://www.w3.org/1999/02/22-rdf-syntax-ns#type | Resource: http://example.org/Domain1 |
| 6. | Resource: http://example.org/bat | RDF Node: http://www.w3.org/2000/01/rdf-schema#range | Resource: http://example.org/Range1 |
| 7. (infered) | Resource: http://example.org/baz2 | RDF Node: http://www.w3.org/1999/02/22-rdf-syntax-ns#type | Resource: http://example.org/Range1 |

  

---

  
**Added the following Triples :**  
<http://example.org/bas> <http://www.w3.org/2000/01/rdf-schema#domain> <http://example.org/Domain2> .  
<http://example.org/bas> <http://www.w3.org/2000/01/rdf-schema#range> <http://example.org/Range2> .
  

|  |  |  |  |
| --- | --- | --- | --- |
| **Base URI:** http://InfModelF.org# | | | **Size:** 11 |
| **No.** | **Subject** | **Predicate** | **Object** |
| 1. | Resource: http://example.org/baz1 | Resource: http://example.org/bat | Resource: http://example.org/baz2 |
| 2. | Resource: http://example.org/bat | RDF Node: http://www.w3.org/2000/01/rdf-schema#subPropertyOf | Resource: http://example.org/bas |
| 3. (infered) | Resource: http://example.org/baz1 | Resource: http://example.org/bas | Resource: http://example.org/baz2 |
| 4. | Resource: http://example.org/bat | RDF Node: http://www.w3.org/2000/01/rdf-schema#domain | Resource: http://example.org/Domain1 |
| 5. (infered) | Resource: http://example.org/baz1 | RDF Node: http://www.w3.org/1999/02/22-rdf-syntax-ns#type | Resource: http://example.org/Domain1 |
| 6. | Resource: http://example.org/bat | RDF Node: http://www.w3.org/2000/01/rdf-schema#range | Resource: http://example.org/Range1 |
| 7. (infered) | Resource: http://example.org/baz2 | RDF Node: http://www.w3.org/1999/02/22-rdf-syntax-ns#type | Resource: http://example.org/Range1 |
| 8. | Resource: http://example.org/bas | RDF Node: http://www.w3.org/2000/01/rdf-schema#domain | Resource: http://example.org/Domain2 |
| 9. (infered) | Resource: http://example.org/baz1 | RDF Node: http://www.w3.org/1999/02/22-rdf-syntax-ns#type | Resource: http://example.org/Domain2 |
| 10. | Resource: http://example.org/bas | RDF Node: http://www.w3.org/2000/01/rdf-schema#range | Resource: http://example.org/Range2 |
| 11. (infered) | Resource: http://example.org/baz2 | RDF Node: http://www.w3.org/1999/02/22-rdf-syntax-ns#type | Resource: http://example.org/Range2 |

  

---

  
**Added the following Triples (loop in the ontology) :**  
<http://example.org/Domain2> <http://www.w3.org/2000/01/rdf-schema#subClassOf> <http://example.org/Domain3> .  
<http://example.org/Domain3> <http://www.w3.org/2000/01/rdf-schema#subClassOf> <http://example.org/Domain2> .

|  |  |  |  |
| --- | --- | --- | --- |
| **Base URI:** http://InfModelF.org# | | | **Size:** 14 |
| **No.** | **Subject** | **Predicate** | **Object** |
| 1. | Resource: http://example.org/baz1 | Resource: http://example.org/bat | Resource: http://example.org/baz2 |
| 2. | Resource: http://example.org/bat | RDF Node: http://www.w3.org/2000/01/rdf-schema#subPropertyOf | Resource: http://example.org/bas |
| 3. (infered) | Resource: http://example.org/baz1 | Resource: http://example.org/bas | Resource: http://example.org/baz2 |
| 4. | Resource: http://example.org/bat | RDF Node: http://www.w3.org/2000/01/rdf-schema#domain | Resource: http://example.org/Domain1 |
| 5. (infered) | Resource: http://example.org/baz1 | RDF Node: http://www.w3.org/1999/02/22-rdf-syntax-ns#type | Resource: http://example.org/Domain1 |
| 6. | Resource: http://example.org/bat | RDF Node: http://www.w3.org/2000/01/rdf-schema#range | Resource: http://example.org/Range1 |
| 7. (infered) | Resource: http://example.org/baz2 | RDF Node: http://www.w3.org/1999/02/22-rdf-syntax-ns#type | Resource: http://example.org/Range1 |
| 8. | Resource: http://example.org/bas | RDF Node: http://www.w3.org/2000/01/rdf-schema#domain | Resource: http://example.org/Domain2 |
| 9. (infered) | Resource: http://example.org/baz1 | RDF Node: http://www.w3.org/1999/02/22-rdf-syntax-ns#type | Resource: http://example.org/Domain2 |
| 10. | Resource: http://example.org/bas | RDF Node: http://www.w3.org/2000/01/rdf-schema#range | Resource: http://example.org/Range2 |
| 11. (infered) | Resource: http://example.org/baz2 | RDF Node: http://www.w3.org/1999/02/22-rdf-syntax-ns#type | Resource: http://example.org/Range2 |
| 12. | Resource: http://example.org/Domain2 | RDF Node: http://www.w3.org/2000/01/rdf-schema#subClassOf | Resource: http://example.org/Domain3 |
| 13. (infered) | Resource: http://example.org/baz1 | RDF Node: http://www.w3.org/1999/02/22-rdf-syntax-ns#type | Resource: http://example.org/Domain3 |
| 14. | Resource: http://example.org/Domain3 | RDF Node: http://www.w3.org/2000/01/rdf-schema#subClassOf | Resource: http://example.org/Domain2 |

  

---

  
**Added the following Triple :**  
<http://example.org/Range3> <http://www.w3.org/2002/07/owl#sameAs> <http://example.org/Range2> .  

|  |  |  |  |
| --- | --- | --- | --- |
| **Base URI:** http://InfModelF.org# | | | **Size:** 16 |
| **No.** | **Subject** | **Predicate** | **Object** |
| 1. | Resource: http://example.org/baz1 | Resource: http://example.org/bat | Resource: http://example.org/baz2 |
| 2. | Resource: http://example.org/bat | RDF Node: http://www.w3.org/2000/01/rdf-schema#subPropertyOf | Resource: http://example.org/bas |
| 3. (infered) | Resource: http://example.org/baz1 | Resource: http://example.org/bas | Resource: http://example.org/baz2 |
| 4. | Resource: http://example.org/bat | RDF Node: http://www.w3.org/2000/01/rdf-schema#domain | Resource: http://example.org/Domain1 |
| 5. (infered) | Resource: http://example.org/baz1 | RDF Node: http://www.w3.org/1999/02/22-rdf-syntax-ns#type | Resource: http://example.org/Domain1 |
| 6. | Resource: http://example.org/bat | RDF Node: http://www.w3.org/2000/01/rdf-schema#range | Resource: http://example.org/Range1 |
| 7. (infered) | Resource: http://example.org/baz2 | RDF Node: http://www.w3.org/1999/02/22-rdf-syntax-ns#type | Resource: http://example.org/Range1 |
| 8. | Resource: http://example.org/bas | RDF Node: http://www.w3.org/2000/01/rdf-schema#domain | Resource: http://example.org/Domain2 |
| 9. (infered) | Resource: http://example.org/baz1 | RDF Node: http://www.w3.org/1999/02/22-rdf-syntax-ns#type | Resource: http://example.org/Domain2 |
| 10. | Resource: http://example.org/bas | RDF Node: http://www.w3.org/2000/01/rdf-schema#range | Resource: http://example.org/Range2 |
| 11. (infered) | Resource: http://example.org/baz2 | RDF Node: http://www.w3.org/1999/02/22-rdf-syntax-ns#type | Resource: http://example.org/Range2 |
| 12. | Resource: http://example.org/Domain2 | RDF Node: http://www.w3.org/2000/01/rdf-schema#subClassOf | Resource: http://example.org/Domain3 |
| 13. (infered) | Resource: http://example.org/baz1 | RDF Node: http://www.w3.org/1999/02/22-rdf-syntax-ns#type | Resource: http://example.org/Domain3 |
| 14. | Resource: http://example.org/Domain3 | RDF Node: http://www.w3.org/2000/01/rdf-schema#subClassOf | Resource: http://example.org/Domain2 |
| 15. | Resource: http://example.org/Range3 | RDF Node: http://www.w3.org/2002/07/owl#sameAs | Resource: http://example.org/Range2 |
| 16. (infered) | Resource: http://example.org/baz2 | RDF Node: http://www.w3.org/1999/02/22-rdf-syntax-ns#type | Resource: http://example.org/Range3 |

  

---

  
**Perform a find(http://example.org/baz1,null,null) :**  

|  |  |  |  |
| --- | --- | --- | --- |
| **Base URI:** http://InfModelF.org# | | | **Size:** 5 |
| **No.** | **Subject** | **Predicate** | **Object** |
| 1. | Resource: http://example.org/baz1 | Resource: http://example.org/bat | Resource: http://example.org/baz2 |
| 2. (infered) | Resource: http://example.org/baz1 | Resource: http://example.org/bas | Resource: http://example.org/baz2 |
| 3. (infered) | Resource: http://example.org/baz1 | RDF Node: http://www.w3.org/1999/02/22-rdf-syntax-ns#type | Resource: http://example.org/Domain1 |
| 4. (infered) | Resource: http://example.org/baz1 | RDF Node: http://www.w3.org/1999/02/22-rdf-syntax-ns#type | Resource: http://example.org/Domain2 |
| 5. (infered) | Resource: http://example.org/baz1 | RDF Node: http://www.w3.org/1999/02/22-rdf-syntax-ns#type | Resource: http://example.org/Domain3 |

  

---

  
**Removed the following Triple :**  
<http://example.org/bat> <http://www.w3.org/2000/01/rdf-schema#subPropertyOf> <http://example.org/bas> .  

|  |  |  |  |
| --- | --- | --- | --- |
| **Base URI:** http://InfModelF.org# | | | **Size:** 11 |
| **No.** | **Subject** | **Predicate** | **Object** |
| 1. | Resource: http://example.org/baz1 | Resource: http://example.org/bat | Resource: http://example.org/baz2 |
| 2. | Resource: http://example.org/bat | RDF Node: http://www.w3.org/2000/01/rdf-schema#domain | Resource: http://example.org/Domain1 |
| 3. | Resource: http://example.org/bat | RDF Node: http://www.w3.org/2000/01/rdf-schema#range | Resource: http://example.org/Range1 |
| 4. | Resource: http://example.org/bas | RDF Node: http://www.w3.org/2000/01/rdf-schema#domain | Resource: http://example.org/Domain2 |
| 5. | Resource: http://example.org/bas | RDF Node: http://www.w3.org/2000/01/rdf-schema#range | Resource: http://example.org/Range2 |
| 6. | Resource: http://example.org/Domain2 | RDF Node: http://www.w3.org/2000/01/rdf-schema#subClassOf | Resource: http://example.org/Domain3 |
| 7. | Resource: http://example.org/Domain3 | RDF Node: http://www.w3.org/2000/01/rdf-schema#subClassOf | Resource: http://example.org/Domain2 |
| 8. | Resource: http://example.org/Range3 | RDF Node: http://www.w3.org/2002/07/owl#sameAs | Resource: http://example.org/Range2 |
| 9. | Resource: http://example.org/Range3 | RDF Node: http://www.w3.org/2002/07/owl#sameAs | Resource: http://example.org/Range2 |
| 10. (infered) | Resource: http://example.org/baz1 | RDF Node: http://www.w3.org/1999/02/22-rdf-syntax-ns#type | Resource: http://example.org/Domain1 |
| 11. (infered) | Resource: http://example.org/baz2 | RDF Node: http://www.w3.org/1999/02/22-rdf-syntax-ns#type | Resource: http://example.org/Range1 |
